# Electron microscopy of the morphological changes in rat viscera during experimental hyperthermic shock

**Published:** 2013-03-25

**Authors:** M Vlad, C Şerboiu, AT Ispas, I Giuvărăşteanu, E Ungureanu, N Ionescu

**Affiliations:** * Department of Clinical Anatomy and Surgical Techniques, Chair of Anatomy; ** Department of Cell and Molecular Biology; *** Department of Anatomy, "Carol Davila" University of Medicine and Pharmacy, Bucharest

**Keywords:** hyperthermic shock, Wistar rats, vital organs, electron microscopy

## Abstract

Hyperthermic shock is a thermoregulatory disorder that affects living organisms that are acutely or chronically exposed to high temperatures or when performing intense physical activity in a hot environment. In this paper, we will show the changes embodied in hyperthermic shock caused by multiple injuries to vital organs in Wistar rats that were suddenly exposed to high temperatures of up to 410 for about 10-15 minutes, their central temperature rising above 40.60C. This process resulted in multiple injuries of the vital organs, evidenced by electron microscopy. In addition, this suggested that most changes caused by hyperthermic shock are incompatible with life.

## Introduction

Numerous cases of morbidity and mortality were observed during exposure to stress in the environment, such as high ambient temperatures. Heat waves caused by global warming may cause changes at the cellular level with consequences on morbidity. A series of animal studies were conducted to observe damage from exposure to heat stress at the cellular and tissue levels that can be associated with increased morbidity and mortality rates. Normal tissues have a low tolerance to sudden exposure to high temperatures. Metabolic stress by hyperthermia is accompanied by a loss of cellular integrity and consequently functional capabilities in all organs. We showed tissue and intracellular distribution of thermal shock injuries in vital organs such as heart, lung, liver and kidney.

## Materials and Methods

 Healthy Wistar rats of 450g body weight had free access to food and water and were kept in temperature-controlled rooms with a 12h light/dark cycle. All animal experiments were carried out in accordance with the international Guidelines for Animal Experimentation. 

 The animals were anesthetized with ether and Ketanest (1 cm³ injected intraperitoneally) and then acutely exposed to a 410C temperature in an incubator. The exposure time ranged between 10–15 minutes until the animals’ movements ceased. The rats were sacrificed by cervical dislocation. Small tissue samples of the heart, lung, liver and kidney were harvested and fined.

Sections (1mm) were processed and Epon embedded for transmission electron microscopy (TEM) as previously described [**14**]. Thin sections of about 60 nm were examined with a Philips 403 transmission electron microscope at 60 kV.

## Results

 Acute exposure of rats to high temperatures for short periods of time determined rapid visceral disorders which might be emphasized through electron microscopy.

 Heart

**Fig. 1:**
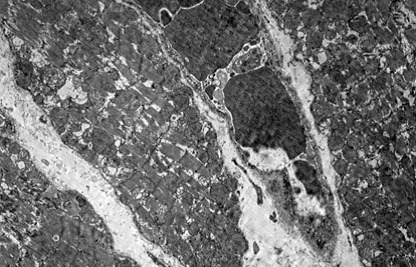
A pronounced interfascicular vascular stasis of the myocardium vessels. The myofibrils are partially altered by mitochondrial and myofilaments damage, 4400×.

**Fig. 2:**
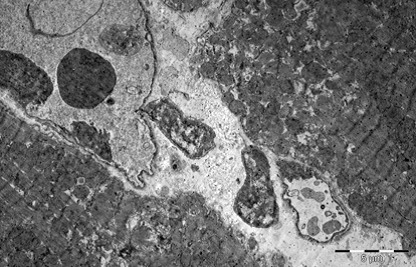
The interfascicular space is enlarged by the interstitial edema. Partial disappearance of the fibrillar structure, 4400×.

**Fig. 3:**
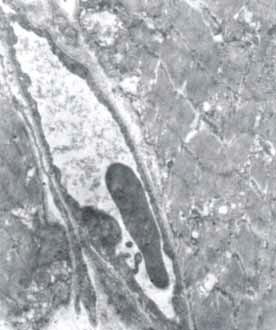
Myocardial fiber fragments separated by conjunctive stroma. Dilated capillary with hematic residue. Partial disappearance of fibrillar structure in myofilaments and mitochondrial disruption, 5600×.

**Fig. 4:**
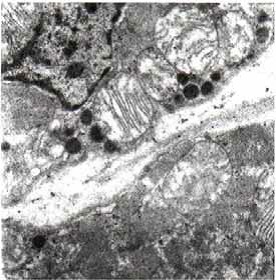
Two fragments of myocardial fiber with altered structure, dilaceration of myofilaments by sarcolemmal edema, dilatation of smooth endoplasmic reticulum, mitochondria with partial blurring and rare vacuolations. Rare electrono-opaque granules of representing natriuretic factor, 7100×

 In myocardium, vascular stasis was marked in interfascicular vessels. Myocardiocytes were partially disorganized. There were frequent mitochondrial injuries. Smooth endoplasmic reticulum was dilated. Interfascicular spaces were enlarged through interstitial edema. In myofilaments, the myofibrils’ structure presented hazy areas. Myofilaments dilacerations have been noticed with the vacuolization of the endoplasmic reticulum. Structure of myofilaments dilacerated through interstitial edema was partially hazy. Intercalary disc was fragmented and fringed. There was a process of condensation of the nuclear material in the myocardiocytic nuclei. Chromatin formed lumps pushed towards the nuclear membrane, which implied the initiation of an apoptotic process. The myocardiocyte degenerative mitochondrial injuries with partial blurring of the cristae and vacuolization of the mitochondrial matrix were accompanied by subsarcolemal appearance of electrono-opaque granules of 300–400 nm in diameter, which contained natriuretic peptide. In the myocardium of the rat, there were large areas of destruction where disorganized mitochondria and atrial natriuretic peptide granules signified the existence of electrolytic metabolic disturbances and severe local ischemic phenomena (**Fig. 1-4**).

 Lung

**Fig. 5:**
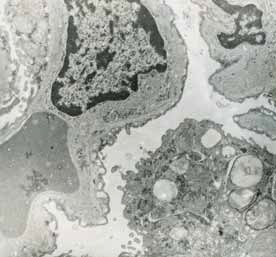
Type II pneumocyte in relation with alveolar capillary. Cell nucleus in apoptosis, 7100×

**Fig. 6:**
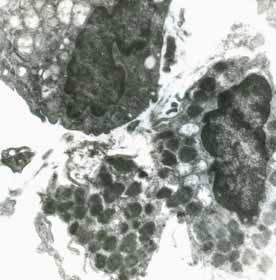
Type II pneumocyte under going degranulation, 7100×

**Fig. 7:**
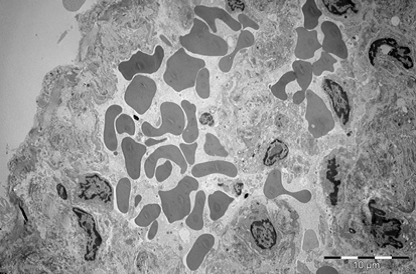
Interstitial extravasated blood with cellular nuclei in apoptosis, 2650×

**Fig. 8:**
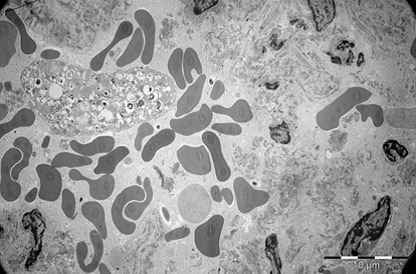
Interstitial extravasated blood with cellular nuclei in apoptosis, 2650×

In the lung, electron microscopy images displayed capillary stasis in the alveolar wall, type II pneumocytes undergoing degranulation and thickening of the interalveolar septa. The lung responded to the hyperthermic stress through capillary hyperemia with the appearance of thrombi, which split the interstitial structure of the lung. Type II pneumocytes suffered an intra-alveolar that were coagulated in blood conglomerates. Apoptotic nuclei were present between the structures separated by blood clots (**Fig. 5-8**).

 Liver

 There were dilated sinusoid capillaries with thrombosis and blood conglomerates in the liver. There were lipid microvacuoles, sign of an early and discrete fat accumulation in the cytoplasm of the hepatocyte. There were vacuolar aspects, which accompanied the partial blurring of the hepatocyte structure. The hepatocyte nucleus was condensed and cellular organelles (mitochondria, smooth endoplasmic reticulum, and rough endoplasmic reticulum) had their structures modified. Cytoplasm suffered a process of destruction and marginalization.

**Fig. 9:**
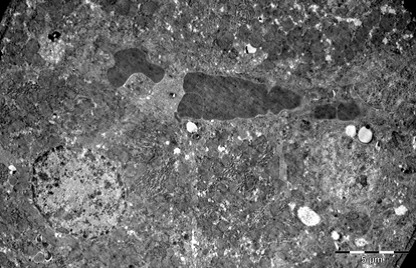
Liver cells fragment with hematic thrombi into the sinusoid capillary, 4400×

**Fig. 10:**
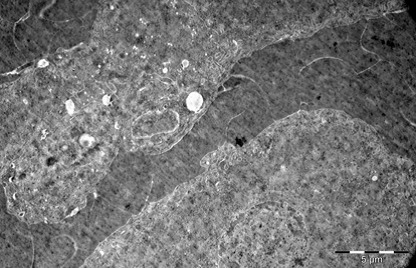
Thrombotic process emphasized in two capillary sinusoids among liver cells, 4400×

 In some areas, mitochondria had a blurred structure through the homogenization of the matrix. Rough endoplasmic reticulum had a structure characterized by intracytoplasmic packages. There were vacuoles of different sizes inside the cytoplasm. The nucleus was visible, with euchromatin and heterochromatin. Blood thrombi occupied the entire capillary lumen, which they enlarged and blocked through confluences. Hepatic microvilli were condensed and they partially occupied the Disse space, which they narrowed (**Fig. 9,10**).

 Kidney

**Fig. 11:**
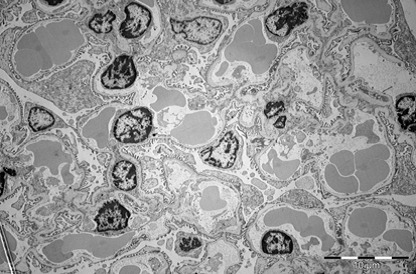
Overview of renal glomeruli with remarkable marked capillary stasis, mesangial cell nucleus in apoptosis. Narrow urinary space, 2650× 4 M. Vlad et al.

**Fig. 12:**
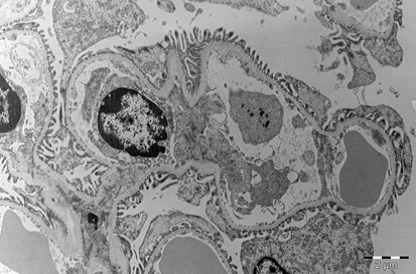
Glomerular zone with narrow urinary space, partial alteration of filtration membrane, marked capillary dilatation with red blood cells and other peripheral blood elements (conglomerates blood platelets), 3400×

**Fig. 13:**
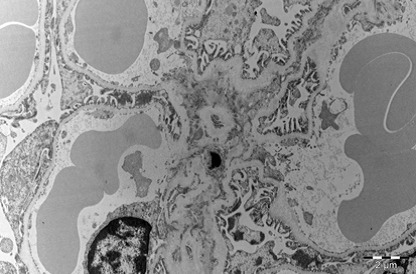
Glomerulus with pronounced alterations of the filtration barrier affecting the endothelium and podocyte extensions. Capillary dilatation with red blood cells conglomerates, 2650×

**Fig. 14:**
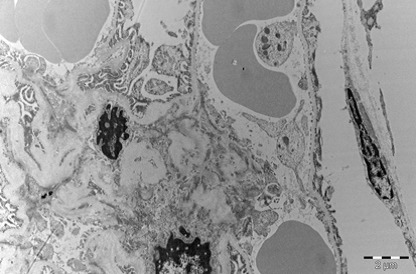
Glomerulus with mesangial cells in apoptosis, destruction of interstitial space with dilated glomerular capillaries by red blood cells and blood platelets, 2650×

**Fig. 15:**
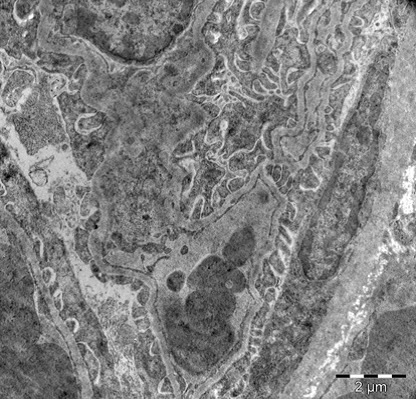
Glomerulus with narrow urinary space due to podocytes proliferation, dilated capillaries and thickening of filtration membrane. Parietal membrane of the glomerulus (PMG), 7100×

**Fig. 16:**
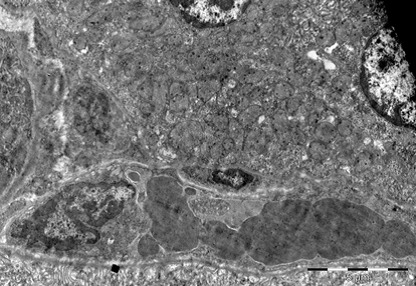
Interstitial capillary cells with red blood cells thrombus among convoluted tubule, 2650×

 During exposure to high temperatures, there was a capillary dilatation in the kidneys, through thrombi accumulation in the stromal vessels situated between the proximal tubules of renal cortex. Blood thrombi from the glomerulus’ capillaries narrowed the urinary space through compression. The long processes of the podocytes were modified. The fenestrated epithelium of the glomerulus’ capillaries was blurred in some areas. There were mitochondrial condensations. Cytoplasm was partially modified. Mesangial cells’ nucleus suffered an apoptotic process. Urinary space was narrowed not only through vascular compression, but also through discrete mesangial proliferation. Renal tubes presented dystrophic modifications at the cortical and medullar level through vascular stasis (**Fig. 11-16**).

## Discussion

 Acute exposure of rats to temperatures increased up to 410C for a limited time determined an increase of the central temperature above 40.60C, followed by multiple visceral lesions, which may be emphasized through electron microscopy [**1-5**]. Hemodynamics alteration in experimental acute hyperthermia appeared during and after exposure to high temperature [**6-7**]. Marked vascular stasis in the myocardium, dilatation of the sinusoid capillaries and blood thrombi from glomerulus capillaries, which narrowed the urinary space, all were characteristics of the hypovolemic shock. Owing to irreversible disturbances of the hemodynamics appear hypoxic secondary lesions in myocardium, lung, kidney and liver parenchyma, which finally determined multiple organ failure [**5,8-13**]. Slowing of the blood circulation disturbed local and general metabolism, affected general homeostasis and compromised the vitality of the tissues lacking oxygen. Following vascular stasis, degenerative vascular lesions appeared, blurring of the glomerulus’ capillaries epithelial fenestrations with the appearance of the edema and involvement of the perivascular tissues. Apoptosis of the myocardiocyte after acute exposure to high temperatures demonstrated that myocardial tissue Electron microscopy of the morphological changes in rat viscera during experimental hyperthermic shock 5, responded fast to stress conditions. Apoptosis in the nuclei of the mesangial cells represented a fast cellular reaction to high temperatures exposure. Dilaceration of the interstitial pulmonary structures was accompanied by nuclear apoptosis.

## Conclusions

The modifications appeared in living beings after thermal shock, which may be emphasized by electron microscopy, are incompatible with life at an important degree.

 Acknowledgements. 

"This paper is supported by the Sectoral Operational Programme Human Resources Development, financed from the European Social Fund and by the Romanian Government under the contract number POSDRU/89/1.5/S/64109" 

 The authors confirm that there are no conflicts of interest
